# Prediction of gold stage in patients hospitalized with COPD exacerbations using blood neutrophils and demographic parameters as risk factors

**DOI:** 10.1186/s12890-021-01696-z

**Published:** 2021-10-21

**Authors:** Jing Chen, Zhao Yang, Qun Yuan, Li-quan Guo, Da-xi Xiong

**Affiliations:** 1 School of Biomedical Engineering (Suzhou), Division of Life Sciences and Medicine, University of Science and Technology of China, Hefei, 230026 People’s Republic of China; 2 Suzhou Institute of Biomedical Engineering and Technology, Chinese Academy of Sciences, Suzhou, 215163 People’s Republic of China; 3 Respiratory Department, The Affiliated Suzhou Science and Technology Town Hospital of Nanjing Medical University, Suzhou, 215163 People’s Republic of China

**Keywords:** GOLD stage, ECOPD, Blood NEU%, Demographic parameter

## Abstract

**Background:**

Patients hospitalized with chronic obstructive pulmonary disease (COPD) exacerbations are unable to complete the pulmonary function test reliably due to their poor health conditions. Creating an easy-to-use instrument to identify the Global Initiative for Chronic Obstructive Lung Disease (GOLD) stage will offer valuable information that assists clinicians to choose appropriate clinical care to decrease the mortality in these patients. The objective of this study was to develop a prediction model to identify the GOLD stage in the hospitalized exacerbation of chronic obstructive pulmonary disease (ECOPD) patients.

**Methods:**

This prospective study involved 155 patients hospitalized for ECOPD. All participants completed lung function tests and the collection of blood neutrophils and demographic parameters. Receiver operating characteristic (ROC) curve was plotted based on the data of 155 patients, and was used to analyze the disease severity predictive capability of blood neutrophils and demographic parameters. A support vector regression (SVR) based GOLD stage prediction model was built using the training data set (75%), whose accuracy was then verified by the testing data set (25%).

**Results:**

The percentage of blood neutrophils (denoted as NEU%) combined with the demographic parameters was associated with a higher risk to severe episode of ECOPD. The area under the ROC curve was 0.84. The SVR model managed to predict the GOLD stage with an accuracy of 90.24%. The root-mean-square error (RMSE) of the forced expiratory volume in one second as the percentage of the predicted value (denoted as FEV_1_%pred) was 8.84%.

**Conclusions:**

The NEU% and demographic parameters are associated with the pulmonary function of the hospitalized ECOPD patients. The established prediction model could assist clinicians in diagnosing GOLD stage and planning appropriate clinical care.

## Background

Chronic obstructive pulmonary disease (COPD) is a progressive lung condition and a leading cause of adult morbidity and mortality worldwide [1–3]. Exacerbation of chronic obstructive pulmonary disease (ECOPD) is an event characterized by a sustained worsening of the respiratory symptoms of a patient (including cough, phlegm production, and dyspnea), beyond normal day-to-day variations, which often necessitates additional therapies [4, 5]. These episodes requiring hospitalization are associated with increased morbidity, mortality, and put enormous burden upon healthcare systems [6, 7]. Inflammation is a key component in the pathogenesis of COPD [8]. It has previously been observed that COPD is not only associated with abnormal inflammatory response of the lung, but also with systemic inflammation, including systemic oxidative stress, activation of circulated immune cells and inflammatory cells, and the increased circulating levels of inflammatory cytokines [9]. It is generally considered that ECOPD reflects a flare-up of these underlying inflammatory processes [10], and is linked to a neutrophilic signature response [11].

A recent systematic literature review concluded that ECOPDs are extremely dangerous events. There is an urgent need to identify tolerable treatment guidelines and manage acute exacerbations in hospitalized ECOPD patients [12]. The Global Initiative for Chronic Obstructive Pulmonary Disease (GOLD) uses the ratio of forced expiratory volume in one second to forced vital capacity (denoted as FEV_1_/FVC) as the diagnostic criteria for airflow obstruction, whose nominal value shall be smaller than 0.70; and classifies the airflow obstruction severity based on the value of forced expiratory volume in one second as percentage of predicted value (denoted as FEV_1_%pred) as shown in Table 1. GOLD stages 1–4 respectively represent mild, moderate, severe and very severe. Inadequate diagnosis of COPD and the lack of spirometric assessment can lead to inadequate treatment strategies, with health costs and risks for patients, leading to delays in diagnosing and treatment of the true cause of the symptoms [13–15]. Patients hospitalized with COPD exacerbations due to poor health status, are unable to complete the pulmonary function test reliably. As one longitudinal study indicated, 50% of pulmonary function test results are unacceptable [16].

**Table 1:** Classification of airflow obstruction severity according to GOLD

In patients with FEV_1_/FVC < 0.70		
GOLD 1	Mild	FEV_1_%pred ≥ 80
GOLD 2	Moderate	50 ≤ FEV_1_%pred < 80
GOLD 3	Severe	30 ≤ FEV_1_%pred < 50
GOLD 4	Very severe	FEV_1_%pred < 30

Many studies on evaluating the disease severity in COPD patients have been focused on the stable stage instead of exacerbating stage. 78% of the ECOPD patients have clear evidence of viral or bacterial infection [17]. The percentage of neutrophils (NEU%) is commonly used as clinical bacterial infection indicators. Based on the needs of unified assessment criteria that can accurately reflect the pulmonary function of hospitalized ECOPD patients, we explored the predictive capability of the percentage of blood NEU% and demographic parameters in GOLD stage and created a prediction model based on support vector regression (SVR) for predicting GOLD stage in Hospitalized ECOPD Patients [18].

## Methods

### Subjects selection

**Fig. 1:**
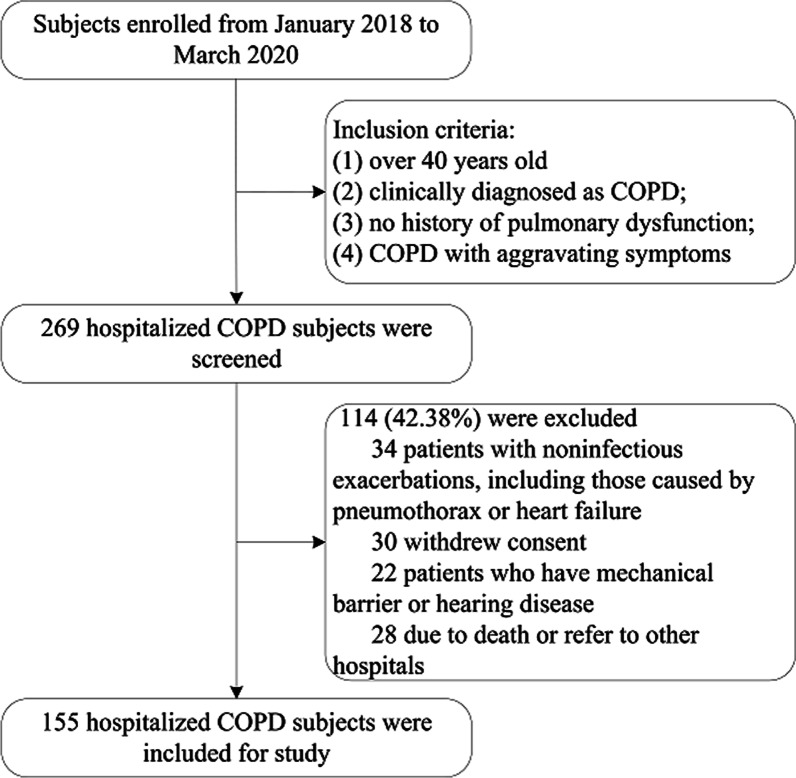
Flowchart of inclusion and exclusion criteria

We conducted a prospective study to explore the predictive capability of the NEU% and demographic parameters in the GOLD stage. A total of 155 subjects (135 males and 20 females) were included in the study, all of whom were from the Respiratory Department of the Affiliated Suzhou Science and Technology Town Hospital of Nanjing Medical University in Suzhou, China. The Medical Ethics Committee approved the study, and all subjects were required to sign an informed consent form. Ethics approval for the data collection and the use of clinical data in the study were obtained from the Ethics Committee of the Affiliated Suzhou Science and Technology Town Hospital of Nanjing Medical University (IRB20180009). 269 candidates were collected from initially selected patients hospitalized for ECOPD. These subjects were over 40 years old, clinically diagnosed as COPD, either with aggravating symptoms or with no history of pulmonary dysfunction. A total of 86 patients were excluded due to the following exclusion criteria: (1) 34 patients with noninfectious exacerbations, including those caused by pneumothorax or heart failure; (2) 30 patients withdrew consent; (3) 22 patients with mechanical barrier or hearing disease; (4) 28 patients due to death or refer to other hospitals. Ultimately, 155 patients were enrolled (Fig. 1).

### Clinical data

**Fig. 2:**
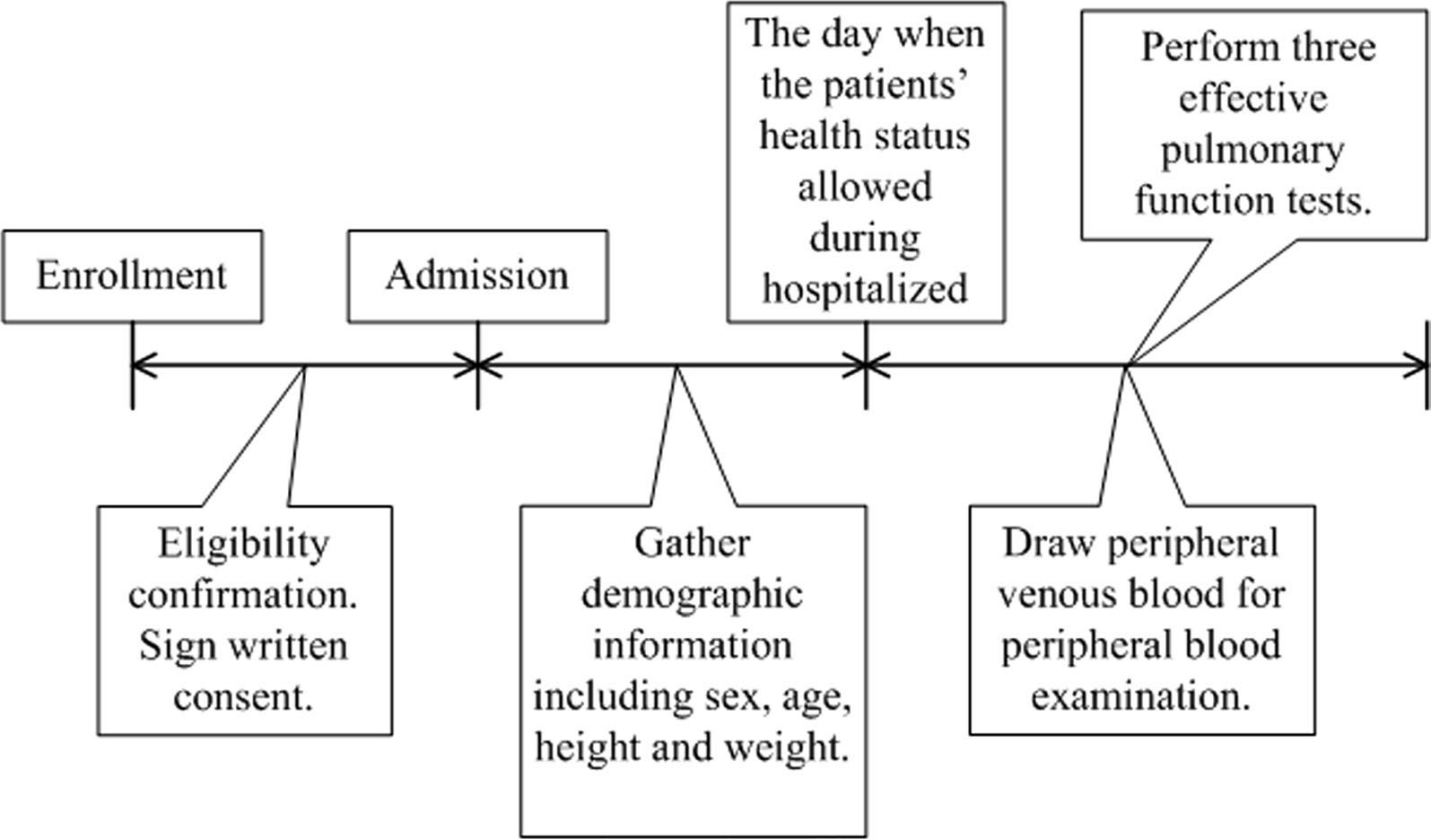
Study timeline

Demographic information including sex, age, height and weight was recorded upon admission to the hospital. After interview and signing a written consent, 155 patients participated in the pulmonary function tests using equipment manufactured by CareFusion, USA. The tests were guided by the same professional doctor when the patients’ health status allowed to do so. Three effective pulmonary function tests were performed with the same, regularly gauged spirometer to reduce measurement errors. The average FEV_1_%pred value and the GOLD stage of the three tests were recorded. FEV_1_ was reported in litres; and the Z-score to correctly characterize the cohorts was investigated. The FEV_1_ Z-score of each patient was derived by using norms from the Global Lung Initiative (GLI) based specially on developed software [19]. Peripheral venous blood was drawn for peripheral blood examination at the same time period with pulmonary function tests. The automatic blood analyzer (SYSMEX Japan) was used to calculate the NEU%. The process from identification of potential patients to data collection can be visualized in Fig. 2.

### Risk stratification

Donner et al. [20] proved that once the FEV1 falls below 1L, it appears that there is a rapid increase in the impact of ECOPD on the daily life and well-being of the patients. Manhire et al. [21] also mentioned in their research that most practicing physicians and radiologists use 1L as a cut off for FEV1 to assess the severity of the disease. We adopted FEV_1_<1L as a threshold to determine the severe episode of ECOPD. All enrolled patients were assessed whether they have severe episodes of ECOPD using FEV_1_<1L as the threshold, and were thus classified into four stages according to GOLD guidelines.

### Statistical analysis

The demographic information, blood NEU% and FEV_1_ of all the participants were expressed as the mean (SD) for normally distributed data or median (IQR) for non-normally distributed data, and the percentages for the categorical variable. Differences of continuous variables between two groups were assessed using Student’s t-tests or Mann-Whitney U tests if normality could not be assumed. Weight and BMI were normally distributed and therefore the Student’s t-tests was used to compare differences between two episode patterns. Mann-Whitney U tests was used for the other variables. Depending on normality assessment of the variables, One-way ANOVA (if normally distributed) or Kruskal-Wallis tests were used to compare differences between more than two groups. Statistical significance was assumed when *P*<0.05. Univariate logistic regression models were developed to assess the correlation between NEU%, demographic parameters and severe episode of ECOPD. All variables correlated with the severe episode of ECOPD were considered in a multivariable model. Receiver operating characteristic (ROC) curves were constructed to evaluate the discrimination of models. An area under the ROC curve of 0.8 or greater is generally considered to be a good predictor [22]. All statistical analyses were performed using SPSS version 24.0.

The outcome variables were defined as the FEV_1_%pred value and the GOLD stage of patients hospitalized for ECOPD. The patients were randomly divided into a training set (75%) and a testing set (25%). The training set was used to develop the SVR based prediction model; whereas the testing set was used to validate the predictive performance of FEV_1_%pred value and GOLD stage. Pearson correlation coefficient was adopted to evaluate the linear correlation of the predicted and the measured values of FEV_1_%pred. If the P-value is less than 0.05, the difference was considered significant. The root mean squared errors (RMSE) and correlation coefficient (r) were used to quantitatively describe the strength of the relationship between the predicted and the measured values of FEV_1_%pred. The GOLD stage was classified by the predicted values of FEV_1_%pred according to the GOLD guidelines, and compared with the GOLD stage classified by the measured value of FEV_1_%pred. The GOLD stage prediction accuracy was also calculated to assess the discrimination capability of the model.

## Results

### Demographic information, blood NEU% and FEV_1_ in all the enrolled patients

Ultimately, 155 subjects were enrolled. A total of 93 subjects were defined as non-severe episode pattern; and 62 subjects were defined as severe episode pattern. The height, weight, BMI, FEV_1_, and FEV_1_%Pred in the non-severe episode group were higher than those in the severe episode group, with significance (*p* < 0.05). Blood NEU% in the non-severe episode group was lower than that in the severe episode group, with significance (*p* < 0.001). There was no significant difference in the sex and age between the two groups (*P* = 0.587, *P* = 0.202) (Table 2).

**Table 2:** Comparison of parameters between non-severe episode group and severe episode group in all enrolled subjects

Category	Variable	Units	FEV_1_ < 1 L as the threshold of severe episode of ECOPD		
			Non-severe episode (N = 93)	Severe episode (N = 62)	*P* value
Demographics	Male	N (%)	82(88.17)	53(85.48)	0.587
	Age	Years	74.00(68.00, 79.00)	76.50(69.00, 80.00)	0.202
	Height	cm	165.50(162.00, 170.00)	163.50(157.50, 168.00)	0.018
	Weight	kg	66.57(9.94)	59.79(10.31)	< 0.001
	BMI		24.38(3.73)	22.65(3.36)	0.008
Blood count	NEU	%	72.05(65.08, 80.95)	82.40(71.80, 86.40)	< 0.001
Pulmonary function	FEV_1_	L	1.37(1.15, 1.72)	0.78(0.67, 0.86)	< 0.001
	FEV_1_ Z-score		-2.29(-2.70, -1.83)	-3.53(-3.77, -3.18)	< 0.001
	FEV_1_%Pred		57.34(49.50, 67.95)	33.46(28.83, 37.46)	< 0.001

Data expressed as mean (SD) or median (IQR); N: numbers of patients

All enrolled patients were classified into GOLD stages 1-4 based on the GOLD. The factors associated with GOLD stage are shown in Table 3. Univariate analysis demonstrated that sex, age, weight, BMI and NEU % are the risk factors of different GOLD stages (Table 3). On the basis of the univariate analysis, the univariable and multivariable models were used to discriminate a severe episode of ECOPD.

**Table 3:** Characteristic distribution comparison among different COPD GOLD stage groups

Characteristic	COPD GOLD stage				P value
	1 (N = 13)	2 (N = 60)	3 (N = 66)	4 (N = 16)	
Sex					0.032
Male	10 (7.41%)	50 (37.04%)	60 (44.44%)	15 (11.11%)	
Female	3 (15%)	11 (55%)	5 (25%)	1 (5%)	
Age					0.001
< 60	2 (15.38%)	10 (76.92%)	1 (7.69%)	0 (0%)	
60–69	4 (7.84%)	24 (47.06%)	18 (35.29%)	5 (9.80%)	
70–79	5 (8.62%)	21 (36.21%)	28 (48.28%)	4 (6.90%)	
≥ 80	2 (6.06%)	6 (18.18%)	18 (54.55%)	7 (21.21%)	
Height					0.101
< 155	2 (16.67%)	7 (58.33%)	3 (25%)	0 (0%)	
155–164	4 (6.67%)	21 (35%)	27 (45%)	8 (13.33%)	
165–174	7 (9.46%)	30 (40.54%)	30 (40.54%)	7 (9.46%)	
≥ 175	0 (0%)	3 (33.33%)	5 (55.56%)	1 (11.11%)	
Weight					0.035
< 50	0 (0%)	5 (26.32%)	13 (68.42%)	1 (5.26%)	
50–59	1 (3.13%)	11 (34.38%)	19 (59.38%)	1 (3.13%)	
60–69	3 (4.84%)	28 (45.16%)	20 (32.26%)	11 (17.74%)	
≥ 70	9 (21.43%)	17 (40.48%)	13 (30.95%)	3 (7.14%)	
BMI					0.038
< 18.5	0 (0%)	6 (30%)	13 (65%)	1 (5%)	
18.5–24.5	3 (4%)	28 (37.33%)	35 (46.67%)	9 (12%)	
24.5–30	9 (17.31%)	23 (44.23%)	14 (26.92%)	6 (11.54%)	
> 30	1 (12.50%)	4 (50%)	3 (37.50%)	0 (0%)	
NEU%					< 0.001
< 70	9 (15.25%)	30 (50.85%)	19 (32.20%)	1 (1.69%)	
70–80	3 (6.98%)	18 (41.86%)	17 (39.53%)	5 (11.63%)	
> 80	1 (1.89%)	13 (24.53%)	29 (54.72%)	10 (18.87%)	

*N* numbers of patients

### Discrimination of a severe episode of ECOPD

**Fig. 3:**
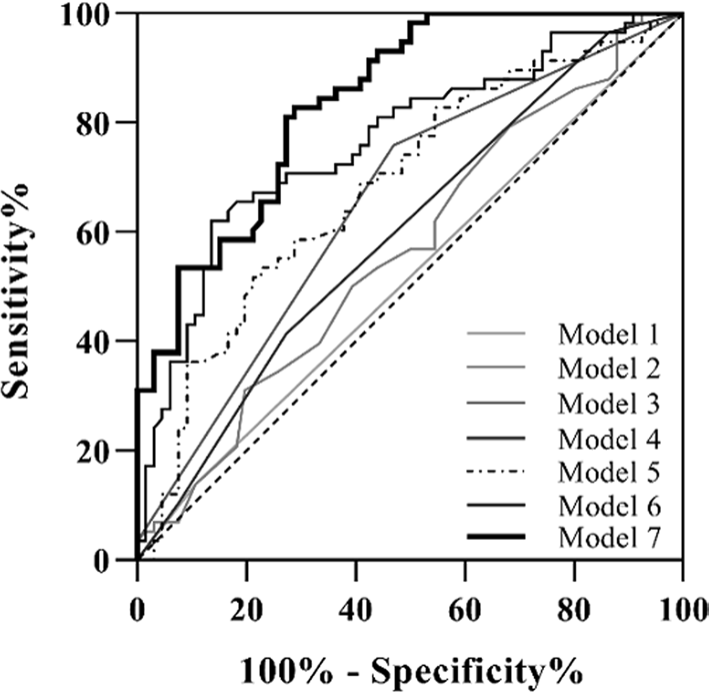
ROC curves for predicting severe episode of ECOPD. The areas under the ROC demonstrate the capability of univariable and multivariable models to predict severe episode of ECOPD. Model 1 = sex; Model 2 = age; Model 3 = weight; Model 4 = BMI; Model 5 = NEU %; Model 6 = multivariable model without NEU%; Model 7 = multivariable model

ROC plots and the areas under the ROC curves of the various models to discriminate a severe episode of ECOPD are shown in Fig. 3 and Table 4. A model incorporating NEU% had an area under the ROC curve of 0.68. The final model including all proved risk factors (Model 7) identified in the multivariable analysis showed good discrimination with an area under the ROC curve of 0.84. The area under the ROC curve of the final model without NEU% (Model 6) was 0.77, indicating that NEU% improved the discrimination accuracy as a useful biomarker.

**Table 4:** The areas under the ROC curves of univariable and multivariable models

Model	The areas under the ROC (95% CI)	*P* value
Model 1	0.52 (0.41 to 0.62)	0.760
Model 2	0.57 (0.47 to 0.67)	0.202
Model 3	0.65 (0.56 to 0.75)	< 0.001
Model 4	0.63 (0.53 to 0.73)	0.013
Model 5	0.68 (0.59 to 0.78)	< 0.001
Model 6	0.77 (0.68 to 0.85)	< 0.001
Model 7	0.84 (0.77 to 0.89)	< 0.001

### GOLD stage prediction

**Fig. 4:**
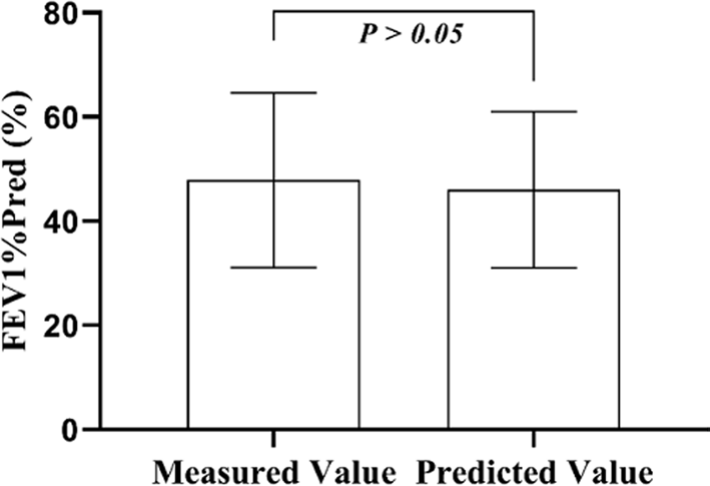
Comparison between the predicted and the measured FEV1%pred values

**Fig. 5:**
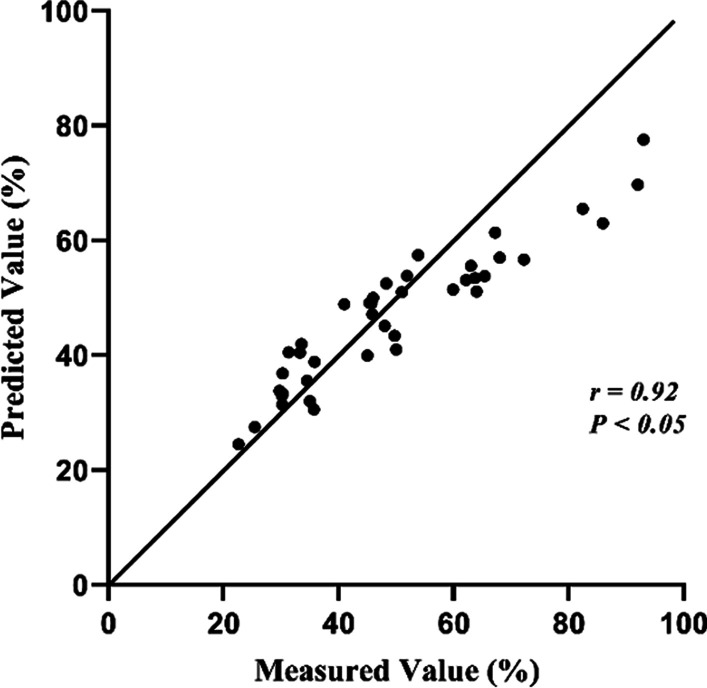
Linear relationship between the predicted and the measured FEV1%pred values

The characteristics of the training set and testing set are included in Table 5. There was no significant difference in all the involved factors, i.e., demographics, blood count, pulmonary function and COPD GOLD stage of subjects between the two groups. Fig. 4 and Fig. 5 show the predictive capability in FEV_1_%pred value of the SVR based prediction model. The association between the predicted and the measured FEV_1_%pred value was strong with r=0.92; and the difference was not significant (*P*>0.05).

**Table 5:** Characteristic of the training set and the testing set

Category	Variable	Units	Training set (N = 114)	Testing set (N = 41)	*P* value
Demographics	Male	N (%)	101 (88.60)	34 (82.93)	0.428
	Age	Years	76.00 (68.00, 80.00)	71.00 (69.00, 78.00)	0.673
	Weight	kg	63.46 (10.15)	63.23 (12.12)	0.917
	BMI	kg/m2	23.54 (3.68)	23.66 (3.63)	0.869
Blood count	NEU	%	76.50 (66.60, 84.25)	78.40 (69.90, 84.30)	0.385
Pulmonary function	FEV_1_%Pred	%	46.59 (34.57, 59.34)	47.66 (32.64, 59.30)	0.822
COPD GOLD Stage	1	N (%)	9 (7.89)	4(9.76)	0.603
	2	N (%)	49 (42.98)	13 (31.72)	
	3	N (%)	43 (37.72)	21 (51.22)	
	4	N (%)	13 (11.40)	3 (7.32)	

N: numbers of patients

**Fig. 6:**
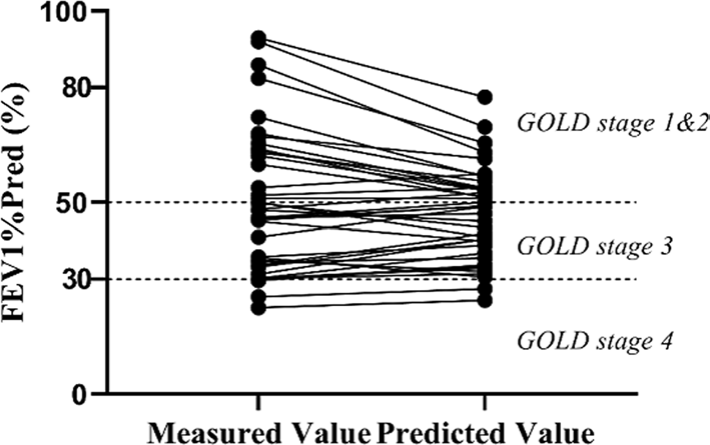
Comparison between the predicted and the measured GOLD stage. GOLD stage 1 and GOLD stage 2 were combined as the moderate group

The total sample size of GOLD stage 1 was only 13, since most hospitalized ECOPD patients tended to have higher GOLD stage. As the degree of airflow limitation of patients with GOLD stage 1 and 2 is moderate, we combined GOLD stage 1 and GOLD stage 2 as the moderate group. The predictive performance on the FEV_1_%pred value and GOLD stage are shown in Table 6. Figure 5 indicated that in the case of FEV_1_%pred exceeding 70%, the model could bring pessimistic prediction results. Analysis on the GOLD stage predictive performance showed that, under the circumstance of predicted FEV_1_%pred exceeding 70%, the algorithm would overestimate the GOLD stage. To be more specific, patients of GOLD stage 1 may be classified to GOLD stage 2. GOLD defines GOLD stage 1 and GOLD stage 2 as moderate airflow obstruction. Their treatment plan will not be confused with GOLD stage 3 and GOLD stage 4, which stand for severe airflow obstruction. Figure 6 shows the predictive performance in different GOLD stage. The overall COPD GOLD stage prediction accuracy was 90.24%.

**Table 6:** Results of the prediction model in the testing sets

Model evaluation		RMSE of FEV_1_% Pred (%)	Accuracy of COPD GOLD stage prediction (%)
Total population of test set (N = 41)		8.84	90.24
*COPD GOLD stage*			
1 and 2 (N = 17)		12.51	94.11
3 (N = 21)		4.98	90.47
4 (N = 3)		2.83	66.67

*N* numbers of patients

## Discussion

ECOPD is a kind of acute attack process, where the patients' respiratory symptoms continue to worsen over their daily status. The frequent episodes of ECOPD resulted in an accelerated decline in FEV_1_. Meanwhile, the rapid decline of FEV_1_ performs as an independent hazard factor for ECOPD. The vicious circle between the decline of FEV_1_ and the frequent attack of ECOPD affect the prognosis and mortality of the patients [23]. In this analysis, we focused on the discrimination value of blood NEU% as a biomarker for a severe episode of ECOPD, and the GOLD stage prediction in hospitalized ECOPD patients. We attempted to create an easy-to-use measure to estimate the value of FEV_1_%Pred and to identify the GOLD stage that could assist clinicians in choosing appropriate measures of medical care to decrease future hospitalization rates and mortality in hospitalized ECOPD patients.

In line with previous studies, the outcome of pulmonary function test relied on the cooperation of ECOPD patients, most likely due to the limitation by force–velocity characteristics of expiratory muscles [16, 24]. Biomarkers were required for effective risk stratification and making individualized treatment decision.

The pathophysiological mechanism of most cases of ECOPD is an acute burst of local or systemic inflammatory mediators following respiratory bacterial or virus infection. Usually, high levels of non-specific inflammatory biomarkers are expected [25]. Neutrophils are the most abundant inflammatory cells in blood and sputum. As neutrophil proteases can generalize many of the characteristics of ECOPD including emphysema and mucus hypersecretion [26], ECOPD is characterized as a neutrophil inflammatory disorder in most cases. A study on peripheral blood neutrophils from ECOPD patients conducted by Milara et. al. showed that compared with healthy control group, the release of the neutrophil activation marker neutrophil elastase (NE) and reactive oxygen species (ROS) increased by 2 times and 30% respectively [27]. Jones et al. observed that compared with the healthy controls, bacteria stimulated neutrophil degranulation was greater in the ECOPD group [28]. Corhay et al. focused on exacerbation whichever its trigger, and found that neutrophil inflammatory markers declined after treatment [29]. We designated a statistically significant difference in the NEU% between ECOPD patients with different GOLD stages to extend these findings. ECOPD patients with higher blood NEU% had a higher tendency of severe episode of ECOPD, whose GOLD stage risk stratification could thus be higher. The differences between ECOPD patients with different GOLD stages are consistent with the results of Perera et al. They found that there were significant differences in systemic markers of inflammation between patients with GOLD stages 3 and 4 vs. controls without COPD; while there was no significant difference between GOLD 2 patients and controls [30].

We sought for factors that would discriminate a severe episode of ECOPD in clinical cases. Although the multivariable demographic parameters or NEU% values reflected the relative risk of a severe episode of ECOPD, considering the moderate values of areas under the ROC curves, the overall prediction performance is still quite limited. No matter which cut-off level is chosen, the false positive rate is still very high, so the specificity for acceptable value of sensitivity is low. With increase in blood NEU%, the risk of a severe episode of ECOPD increased. The overall discrimination value of multivariable factors including demographic parameters and blood NEU% was encouraging with the area under the ROC curve of 0.84.

To further study the FEV_1_%Pred prediction and the GOLD stage categorization capability of the blood NEU% and demographic parameters, we randomly divided the data collected from the ECOPD patients into a training data set to develop a prediction model and a testing data set to validate the predictive performance. The selected demographic parameters included sex, age, weight and BMI, which had demonstrated their relevance to the target values. We used supervised learning algorithm to evaluate the predictive capability of the risk factors, and classified the subjects to 4 different GOLD stages. Searching for the right subjects was one of the major difficulties of our study.

On the other hand, support vector machine (SVM) is a learning method based on the principle of structural risk minimization of statistical learning theory. It shows many unique advantages in solving the problem of small sample and nonlinearity [18]. SVR is a model dealing with the SVM regression problems, which showed acceptable regression capacity in estimating the value of FEV_1_%Pred and identifying the GOLD stage.

To our knowledge, this is the first study in ECOPD patients to predict the value of FEV_1_%Pred and identify the GOLD stage based on demographic parameters and blood NEU%. In the absence of a clear biomarker to categorize the GOLD stage of ECOPD patients, our research provides an auxiliary guidance value for the clinicians to diagnose GOLD stage and establish appropriate clinical care, since the demographic parameters and blood NEU% are easy to be obtained.

Limitations of our current study should also be noted. First, the relatively small number of subjects enrolled in this study could limit the predictive performance of the model, especially when comparing to the previous work of Cristóbal et al. [31] and Godtfredsen and coworkers [32]. The predictive performance of the prediction model was limited in the ECOPD patients with optimistic degree of airflow obstruction, which could also be resulted from the lower influence of inflammatory factors when the symptoms were moderate. To find proper ECOPD patients and guide them to complete the pulmonary function test turned out to be one of the biggest difficulties during our research. To overcome this limitation, we used the most widely accepted learning method SVM to establish the prediction model. The grouping strategy of the training set and testing set was able to tackle the problem of multiple covariates larger than the samples (patients) or “p > n problem”. Importantly, the overall ECOPD GOLD stage prediction accuracy of the establish prediction model was 90.24%. Besides, Sørheim and coworkers showed that pulmonary function injury may differ between sexes. There was a sexual imbalance in our study, as the ECOPD patients included were mostly male (135/155). The model’s predictive performance on female patients could be limited. Considering the low population of the study, comorbidity and different treatments during hospitalization that are not reported herein, could influence the result of this work. Therefore, our future work is to balance the sex composition and extend the observation time to carry out larger scale research to verify our findings. As an additional limitation of the study, the patient's general condition, comprehension and cooperative degree could also influence the accuracy of pulmonary function test results. Nevertheless, every enrolled patient was trained and guided by the same professional physician to minimize the impact of external factors on the measurement.

## Conclusions

In summary, a prediction model based on demographic parameters and blood NEU% has been established to predict the value of FEV_1_%Pred and identify the GOLD stage of the patients hospitalized with ECOPD. This easy-to-use instrument can assist clinicians in diagnosing GOLD stage, and offers valuable information to determine the appropriate clinical care for hospitalized ECOPD patients.

## Data Availability

The datasets used and/or analyzed during the current study are available from the corresponding author on reasonable request.

## Abbreviations

COPD: Chronic Obstructive Pulmonary Disease
GOLD: The Global Initiative for Chronic Obstructive Lung Disease
ECOPD: Exacerbation of Chronic Obstructive Pulmonary Disease
ROC: The Receiver Operating Characteristic
SVR: Support Vector Regression
NEU%: The Percentage of Neutrophils
RMSE: The Root-mean-square Error
FEV_1_/FVC: The Ratio of Forced Expiratory Volume in One Second to Forced Vital Capacity
FEV_1_%pred: The Forced Expiratory Volume in One Second as the Percentage of the Predicted
GLI: The Global Lung Initiative
ROS: Reactive Oxygen Species
NE: Neutrophil Elastase
SVM: Support Vector Machine
